# Low Intensity Physical Exercise Attenuates Cardiac Remodeling and Myocardial Oxidative Stress and Dysfunction in Diabetic Rats

**DOI:** 10.1155/2015/457848

**Published:** 2015-10-05

**Authors:** C. Gimenes, R. Gimenes, C. M. Rosa, N. P. Xavier, D. H. S. Campos, A. A. H. Fernandes, M. D. M. Cezar, G. N. Guirado, A. C. Cicogna, A. H. R. Takamoto, M. P. Okoshi, K. Okoshi

**Affiliations:** ^1^Department of Internal Medicine, Botucatu Medical School, São Paulo State University (UNESP), S/N, Rubião Junior District, 18618 970 Botucatu, SP, Brazil; ^2^Sagrado Coração University, Bauru, SP, Brazil; ^3^Department of Chemistry and Biochemistry, Institute of Biosciences, São Paulo State University (UNESP), Brazil

## Abstract

We evaluated the effects of a low intensity aerobic exercise protocol on cardiac remodeling and myocardial function in diabetic rats. Wistar rats were assigned into four groups: sedentary control (C-Sed), exercised control (C-Ex), sedentary diabetes (DM-Sed), and exercised diabetes (DM-Ex). Diabetes was induced by intraperitoneal injection of streptozotocin. Rats exercised for 9 weeks in treadmill at 11 m/min, 18 min/day. Myocardial function was evaluated in left ventricular (LV) papillary muscles and oxidative stress in LV tissue. Statistical analysis was given by ANOVA or Kruskal-Wallis. Echocardiogram showed diabetic groups with higher LV diastolic diameter-to-body weight ratio and lower posterior wall shortening velocity than controls. Left atrium diameter was lower in DM-Ex than DM-Sed (C-Sed: 5.73 ± 0.49; C-Ex: 5.67 ± 0.53; DM-Sed: 6.41 ± 0.54; DM-Ex: 5.81 ± 0.50 mm; *P* < 0.05 DM-Sed vs C-Sed and DM-Ex). Papillary muscle function was depressed in DM-Sed compared to C-Sed. Exercise attenuated this change in DM-Ex. Lipid hydroperoxide concentration was higher in DM-Sed than C-Sed and DM-Ex. Catalase and superoxide dismutase activities were lower in diabetics than controls and higher in DM-Ex than DM-Sed. Glutathione peroxidase activity was lower in DM-Sed than C-Sed and DM-Ex. *Conclusion.* Low intensity exercise attenuates left atrium dilation and myocardial oxidative stress and dysfunction in type 1 diabetic rats.

## 1. Introduction

Diabetes mellitus is an important public health issue due to its high prevalence and increased morbidity and mortality. Cardiovascular disease is a major cause of death in diabetic patients [1]. Cardiac injury is caused by coronary atherosclerosis and diabetes-related cardiomyopathy [1]. As first reported by Rubler et al. [2], diabetic cardiomyopathy is a single form of heart disease characterized by left ventricular systolic and diastolic dysfunction in the absence of underlying coronary artery disease and/or hypertension. Diabetic cardiomyopathy is a common cardiac condition affecting both type 1 and type 2 diabetes patients [3].

The pathophysiology of diabetic cardiomyopathy is not completely understood as several mechanisms can be involved; these include myocyte hypertrophy, myocardial fibrosis, contractile dysfunction, calcium handling and mitochondrial function changes, and nitric oxide signaling impairment [3–8]. Hyperglycemia-induced oxidative stress is an important factor involved in diabetic cardiomyopathy [9–12].

Regular physical exercise is an established nonpharmacological strategy used as an adjuvant therapy in heart failure from different etiologies [13, 14]. Clinical studies in stable chronic heart failure have shown that long-term moderate physical training attenuates abnormal cardiac remodeling and improves functional capacity, exercise duration, and quality of life [15–18]. In different cardiac injury models, exercise has been shown to attenuate left ventricular dilatation, myocyte hypertrophy, myocardial fibrosis, mitochondrial dysfunction, myocyte calcium handling changes, sympathoexcitation alterations, cardiac dysfunction, and inflammatory activation [19–25].

In diabetes, physical exercise reduces cardiovascular risk factors and improves glycemic control, functional capacity, and muscle strength [26–29]. However, most clinical studies in diabetes have been performed on type 2 diabetes patients [28, 30]. In experimental studies on rats with streptozotocin-induced diabetes, regular physical exercise has been shown to improve myocardial glucose homeostasis, endogenous antioxidant defenses, cardiac function, heart tolerance to ischemia, and ultrastructural extracellular matrix and mitochondrial changes [31–33].* In vivo* evaluation of cardiac function is subjected to myocardial function modulation by hemodynamic and systemic metabolic abnormalities. Left ventricular isolated papillary muscle preparations allow us to properly control preload and afterload and analyze intrinsic myocardial function without the effects of systemic metabolic changes [34–36]. Furthermore, by using positive inotropic stimulation, it is also possible to evaluate myocardial contractile reserve in papillary muscle preparations [37, 38]. Therefore, in this study we evaluated the influence of a low intensity aerobic exercise protocol on* in vivo* cardiac remodeling and* in vitro* myocardial function in rats with streptozotocin-induced diabetes mellitus. As oxidative stress is associated with diabetes cardiomyopathy and can be influenced by physical exercise, we also analyzed myocardial oxidative stress in diabetic rats.

## 2. Methods

Male Wistar rats were purchased from the Central Animal House at Botucatu Medical School, UNESP. All animals were housed in a room under temperature control at 23°C and kept on a 12-hour light/dark cycle. Food and water were supplied* ad libitum*. All experiments and procedures were approved by Botucatu Medical School Ethics Committee, UNESP, Botucatu, SP, Brazil.

The rats were assigned into four groups: sedentary control (C-Sed, *n* = 14); exercised control (C-Ex, *n* = 15); sedentary diabetes (DM-Sed, *n* = 25); and exercised diabetes (DM-Ex, *n* = 25). Diabetes was induced by intraperitoneal injection of streptozotocin (Sigma, St. Louis, MO, USA) at the dose of 50 mg/kg diluted in 0.01 M citrate buffer pH 4.5 [9, 39–41]. Seven days after streptozotocin administration, blood glucose was measured by glucometer (advantage); only rats with glycemia > 220 mg/dL were considered diabetic and included in the study [9].

After diabetes confirmation, exercise protocol was started in a treadmill (AVS Projects, São Paulo, Brazil) as previously described [42]. The protocol was applied once a day, five days a week, for nine weeks. After the first week of animals adaptation (8 min, 8 m/min), exercise duration and speed were increased gradually up to 18 min/day at 11 m/min. In the first two weeks of training, the animals were subjected to low-voltage electrical stimulation to start the exercise. No animals were lost during exercise training.

Systolic blood pressure was measured at the end of experiment by the tail-cuff method [36] using an electrosphygmomanometer (*Narco Bio-System*, model 709-0610, International Biomedical Inc., USA).

### 2.1. Echocardiographic Study

Echocardiographic evaluation was performed using a commercially available echocardiograph (General Electric Medical Systems, Vivid S6, Tirat Carmel, Israel) equipped with a 5–11.5 MHz multifrequency probe. Rats were anesthetized by intraperitoneal injection of a mixture of ketamine (50 mg/kg) and xylazine (0.5 mg/kg). A two-dimensional parasternal short-axis view of the left ventricle (LV) was obtained at the level of the papillary muscles. M-mode tracings were obtained from short-axis views of the LV at or just below the tip of the mitral-valve leaflets and at the level of the aortic valve and left atrium [43–46]. M-mode LV images were printed on a black-and-white printer at a sweep speed of 200 mm/s. All LV structures were manually measured by the same observer (KO) according to the leading-edge method of the American Society of Echocardiography. The measurements obtained were the mean of at least five cardiac cycles on the M-mode tracings. The following structural variables were measured: left atrium (LA) diameter, LV diastolic and systolic diameters (LVDD and LVSD, resp.), LV diastolic posterior wall thickness (PWT), and aortic diameter (AO). LV relative wall thickness (RWT) was calculated by the following formula: 2 × PWT/LVDD. Left ventricular mass (LVM) was calculated using the following formula: [(LVDD + PWT + SWT)³  − (LVDD)³] × 1.04. Left ventricular function was assessed by the following parameters: endocardial fractional shortening (EFS), ejection fraction (EF), posterior wall shortening velocity (PWSV), early-to-late diastolic mitral inflow velocities ratio (E/A ratio), and E wave deceleration time (EDT). A joint assessment of diastolic and systolic LV function was performed by the myocardial performance index (Tei index).

### 2.2. Myocardial Functional Analysis

Two days after the echocardiographic study, intrinsic myocardial contractile performance was evaluated in isolated LV papillary muscle preparation as previously described [47–50]. Rats were anesthetized (sodium pentobarbital intraperitoneally, 50 mg/kg) and decapitated. Hearts were quickly removed and placed in oxygenated Krebs-Henseleit solution at 28°C. LV anterior or posterior papillary muscle was dissected free, mounted between two spring clips, and placed vertically in a chamber containing Krebs-Henseleit solution at 28°C and oxygenated with a mixture of 95% O_2_ and 5% CO_2_ (pH 7.38). The composition of the Krebs-Henseleit solution in mM was as follows: 118.5 NaCl, 4.69 KCl, 1.25 CaCl_2_, 1.16 MgSO_4_, 1.18 KH_2_PO_4_, 5.50 glucose, and 25.88 NaHCO_3_. The spring clips were attached to a Kyowa model 120T-20B force transducer and a lever system which allowed for muscle length adjustment. Preparations were stimulated 12 times/min at a voltage 10% above threshold.

After a 60-minute period, during which the preparations were permitted to shorten whilst carrying light loads, muscles were loaded to contract isometrically and stretched to the apices of their length-tension curves (*L*_max⁡_). After a 5-minute period, during which preparations performed isotonic contractions, muscles were again placed under isometric conditions and length-tension curve apex was determined. A 15-minute period of stable isometric contraction was imposed prior to the experimental period. One isometric contraction was then recorded for later analysis.

The following parameters were measured from isometric contraction: peak of developed tension (DT, g/mm^2^), resting tension (RT, g/mm^2^), time to peak of tension (TPT, ms), maximum rate of tension development (+dT/dt, g/mm^2^/s), and maximum rate of tension decline (−dT/dt, g/mm^2^/s). To evaluate contractile reserve, mechanical papillary muscle performance was evaluated at basal condition and after the following inotropic stimulation: postrest contraction, extracellular Ca^2+^ concentration increase, and beta-adrenergic agonist isoproterenol addition to the nutrient solution [36].

Papillary muscle cross-sectional area (CSA) was calculated from muscle weight and length by assuming cylindrical uniformity and a specific gravity of 1.0. All force data were normalized for muscle CSA. Papillary muscles with CSA > 1.7 mm^2^ were excluded from analysis as muscles with CSA > 1.7 mm^2^ can present central core hypoxia and impaired functional performance.

After dissecting papillary muscle, atria and ventricles were separated and weighed. Fragments of lung and left and right ventricles were weighed before and after drying sessions (65°C for 72 h) to evaluate the wet-to-dry weight ratio.

### 2.3. Morphologic Analysis

Transverse LV sections were fixed in 10% buffered formalin and embedded in paraffin. Five-micrometer-thick sections were stained with hematoxylin and eosin. From each LV, the smallest transverse diameter was measured in at least 50 myocytes, in which the nucleus could be clearly identified [9]. Measurements were performed using a Leica microscope (magnification 40x) attached to a video camera and connected to a computer equipped with image analysis software (Image-Pro Plus 3.0, Media Cybernetics, Silver Spring, MD, USA).

### 2.4. Myocardial Hydroxyproline

To estimate myocardial collagen content, hydroxyproline concentration was measured in LV tissue as previously described [51, 52]. Briefly, the tissue was dried using a SpeedVac Concentrator SC 100 attached to a refrigerated condensation trap (TRL 100) and vacuum pump (VP 100, Savant Instruments, Inc., Farmingdale, NY, USA). Tissue dry weight was measured and the samples were hydrolyzed overnight at 100°C with 6 N HCl (1 mL/10 mg dry tissue). A 50 *μ*L aliquot of the hydrolysate was dried in the SpeedVac Concentrator; 1 *μ*L of deionized water was added; and the sample transferred to a Teflon tube. One milliliter of potassium borate buffer (pH 8.7) was added to maintain constant pH and the sample was oxidized with 0.3 mL of chloramine T solution at room temperature for 20 min. The addition of 1 mL of 3.6 M sodium thiosulfate and thorough mixing for 10 s stopped the oxidative process. The solution was saturated with 1.5 g KCl. The tubes were capped and heated in boiling water for 20 min. After cooling to room temperature, the aqueous layer was extracted with 2.5 mL of toluene; 1.5 mL of toluene extract was transferred to a tube; and 0.6 mL of Ehrlich's reagent was added. The color was allowed to develop for 30 min. Absorbance was read at 565 mm against a reagent blank. Deionized water and 20 *μ*g/mL HOP were used as the blank and standard, respectively.

### 2.5. Serum Biochemical Analysis

Biochemical parameters were measured in serum using a spectrophotometer from Pharmacia Biotech (Ultrospec 2000, Cambridge, England). Analyses were performed with a CELM kit (Modern Laboratory Equipment Company, São Paulo, Brazil). Glycemia was determined by enzymatic method using glucose oxidase and peroxidase. Total cholesterol levels were measured enzymatically using cholesterol ester/oxidase. High-density lipoprotein (HDL) concentration was measured after precipitation of VLDL and LDL by the sodium phosphotungstate/Mg^++^ method using an enzymatic colorimetric method that incorporates polyethylene glycol-modified cholesterol ester oxidase.

### 2.6. Myocardial Oxidative Stress

Oxidative stress was assessed as previously described [9, 53]. Myocardial lipid hydroperoxide concentration was measured in medium containing methanol 90% (v/v), 250 *μ*M ammonium ferrous sulfate, 100 *μ*M xylenol orange, 25 mM sulfuric acid, and 4 mM butylated hydroxytoluene. The solution was incubated for 30 min at room temperature and measured at 560 nm. Glutathione peroxidase was assayed in 15 *μ*L using 0.15 M phosphate buffer, pH 7.0, containing 5 mM EDTA, 0.1 mL of 0.0084 M NADPH, 4 *μ*g of glutathione-reductase, 1.125 M sodium azide, and 0.15 M glutathione reduced form (GSH) in a total volume of 0.3 mL. Superoxide dismutase (SOD) activity was determined using its ability to inhibit reduction of nitroblue tetrazolium, in medium containing 50 mM phosphate buffer pH 7.4, 0.1 mM EDTA, 50 *μ*M NBT, 78 *μ*M NADH, and 3.3 *μ*M phenazine methosulfate. One unit of SOD was defined as the amount of protein needed to decrease the reference rate to 50% of maximum inhibition. Enzyme activities were analyzed at 25°C using a microplate reader system (*μ*Quant-MQX 200 with KC Junior software, Bio-Tek Instruments, Winooski, VT, USA). Catalase activity was determined in a mixture containing 10 mM hydrogen peroxide and sodium phosphate buffer 50 mM, pH 7.0, in a final volume of 0.3 mL. Catalase unit was defined as the amount of enzyme related to *μ*mol H_2_O_2_ decomposition in a first order rate constant during 15 s, at 240 nm. All reagents were purchased from Sigma (St. Louis, MO, USA).

### 2.7. Statistical Analysis

Values were expressed as mean ± standard deviation or median and 25th and 75th percentiles, according to normal or nonnormal distribution, respectively. Parameters with normal distribution were compared by one way ANOVA followed by the Bonferroni post hoc test and nonnormal parameters were compared by Kruskal-Wallis and the Dunn post hoc test (comparisons of interest: C-Ex versus C-Sed, DM-Sed versus C-Sed, and DM-Ex versus DM-Sed). Systat 12 for Windows software was used for analyses. Statistical significance was accepted at the level of *P* < 0.05.

## 3. Results

**Figure 1:**
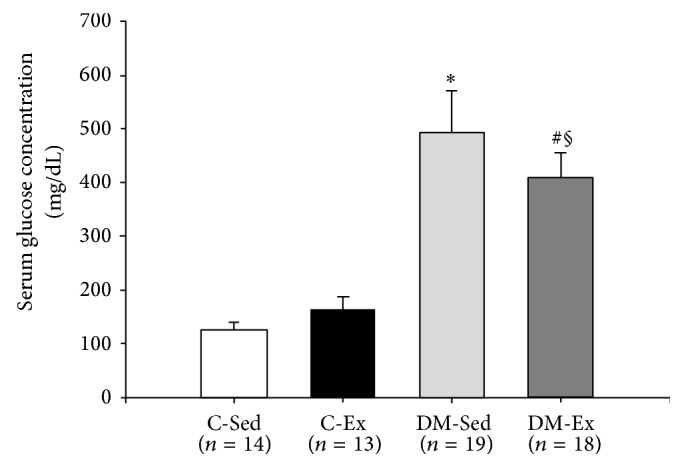
Serum glucose concentration at the end of the experiment. C-Sed: sedentary control group; C-Ex: exercised control group; DM-Sed: sedentary diabetic group; DM-Ex: exercised diabetic group. ANOVA and Bonferroni; ^∗^ *P* < 0.05 versus C-Sed; ^#^ *P* < 0.05 versus C-Ex; ^§^ *P* < 0.05 versus DM-Sed.

The final number of animals in each experimental group was 14 in C-Sed, 13 in C-Ex, 19 in DM-Sed, and 18 in DM-Ex. Fifteen rats were excluded from the study due to nonadaptation to treadmill exercise or failure to increase glycemia after streptozotocin administration. Initial body weight (C-Sed: 327 ± 23; C-Ex: 331 ± 13; DM-Sed: 336 ± 20; DM-Ex: 340 ± 20 g; *P* > 0.05), glycemia (C-Sed: 109 ± 11; C-Ex: 108 ± 14; DM-Sed: 103 ± 12; DM-Ex: 102 ± 12 mg/dL; *P* > 0.05), and final systolic blood pressure (C-Sed: 131 ± 18; C-Ex: 126 ± 13; DM-Sed: 135 ± 18; DM-Ex 140 ± 22 mmHg; *P* > 0.05) did not differ between groups. Final glycemia was higher in diabetic groups than controls and slightly lower in DM-Ex than DM-Sed (Figure 1).

Table 1 presents anatomical data. Final body weight was lower in diabetic groups than controls and in exercised versus sedentary groups. LV and right ventricle weights were lower in diabetic groups than their respective controls. However, LV-to-body weight ratio was higher in DM than control groups. Atria weight was lower in DM-Ex than C-Ex and DM-Sed.

Table 2 shows echocardiographic cardiac structural and left ventricular functional parameters. Heart rate did not differ between groups. C-Ex presented lower LV diastolic diameter and higher relative wall thickness than C-Sed. Diabetic groups had higher LV diastolic diameter-to-body ratio, left atrial diameter-to-aorta diameter, and LV mass index and lower posterior wall shortening velocity than control groups. Left atrium diameter, in absolute or normalized to aorta diameter values, was lower in DM-Ex than in DM-Sed.

Table 3 shows LV papillary muscle functional data. In basal condition, +dT/dt was lower in C-Ex than C-Sed. After postrest contraction, +dT/dt was lower in C-Ex and DM-Sed than C-Sed; DT and −dT/dt were lower in DM-Sed than C-Sed. After extracellular calcium concentration increase to 2.5 mM, TPT was greater in DM-Sed than C-Sed. After isoproterenol addition, DM-Sed presented increased TPT and reduced +dT/dt and −dT/dt compared to C-Sed; DM-Ex had lower −dT/dt than C-Ex.

Myocardial hydroxyproline concentration (C-Sed: 2.06 ± 0.50; C-Ex: 1.96 ± 0.09; DM-Sed: 2.19 ± 0.40; DM-Ex: 2.29 ± 0.36 mg/g; *P* > 0.05) and LV myocyte diameter (C-Sed: 12.7 ± 0.90; C-Ex: 13.8 ± 1.62; DM-Sed: 13.1 ± 1.08; DM-Ex: 13.7 ± 0.69 *μ*m; *P* > 0.05) did not differ between groups. Total cholesterol concentration was higher in both DM groups than controls and lower in DM-Ex than DM-Sed (C-Sed: 90.5 (86.5–100); C-Ex: 98 (86.5–121); DM-Sed: 188 (182–198); DM-Ex: 148 (136–165) mg/dL; *P* < 0.05). HDL-cholesterol was lower in DM-Sed than C-Sed and DM-Ex (C-Sed: 41.6 (37.8–49.0); C-Ex: 38.6 (37.1–45.3); DM-Sed: 21.5 (20.8–24.5); DM-Ex: 40.8 (30.4–43.8) mg/dL; *P* < 0.05).

**Figure 2:**
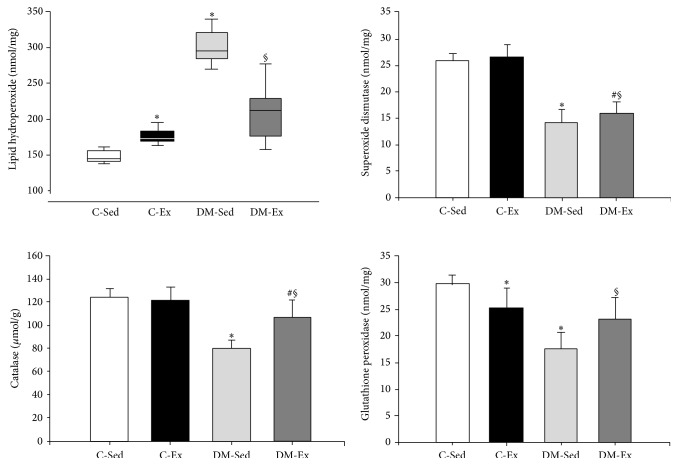
Oxidative stress markers assessed in the left ventricular myocardium. C-Sed: sedentary control group; C-Ex: exercised control group; DM-Sed: sedentary diabetic group; DM-Ex: exercised diabetic group. ANOVA and Bonferroni or Kruskal-Wallis and Dunn (lipid hydroperoxide concentration); ^∗^ *P* < 0.05 versus C-Sed; ^#^ *P* < 0.05 versus C-Ex; ^§^ *P* < 0.05 versus DM-Sed.

Oxidative stress data are shown in Figure 2. Lipid hydroperoxide concentration was higher in C-Ex and DM-Sed than C-Sed and lower in DM-Ex than DM-Sed. Catalase and superoxide dismutase activity was lower in DM groups than their respective controls and higher in DM-Ex than DM-Sed. Glutathione peroxidase activity was lower in C-Ex and DM-Sed than C-Sed and higher in DM-Ex than DM-Sed.

## 4. Discussion

In this study, we evaluated the effects of a low intensity physical exercise protocol on cardiac remodeling and myocardial oxidative stress and function in type 1 diabetic rats.

We used a low intensity exercise protocol for nine weeks including one week of adaptation. Low intensity exercise protocols have been used in diabetic rats as they may not tolerate more extensive or intense periods of exercise [42, 54, 55]. Low intensity appeared more effective than high intensity exercise training to reduce diabetes-related inflammation in serum and skeletal muscles [56]. In our study, the low intensity exercise protocol was sufficient to reduce myocardial oxidative stress and to improve cardiac remodeling and myocardial function.

As expected, diabetic animals presented heightened glycemia increase and reduced body weight and physical exercise slightly attenuated serum glucose increase in DM-Ex group. As blood pressure did not differ between groups, we can discard the influence of hemodynamic changes on exercise-induced cardiac changes.

Transthoracic echocardiogram was performed to analyze* in vivo* cardiac structures and left ventricular function. In the C-Ex group, physical exercise induced a slight reduction in absolute LV diastolic diameter values and an increase in LV relative wall thickness with unchanged systolic or diastolic function. These results suggest a change towards LV concentric remodeling.

DM-Sed presented LV hypertrophy with dilated left cardiac chambers, characterized by increased LV mass index, LV diastolic diameter-to-body weight ratio, and left atrial diameter, with reduced systolic function, characterized by decreased LV posterior wall shortening velocity, compared to C-Sed. A similar echocardiographic pattern of dilated cardiomyopathy has often been observed in experimental diabetes mellitus [57–61]. The fact that posterior wall shortening velocity was the only changed functional variable suggests that cardiac function was evaluated early during diabetes cardiomyopathy development. The higher lung wet-to-dry weight ratio in DM-Sed suggests the presence of pulmonary congestion and reinforces the LV dysfunction in DM-Sed. Myocardial function analysis in LV papillary muscle preparations showed lower developed tension in postrest contraction; lower +dT/dt in basal condition, postrest contraction, and after isoproterenol stimulation; lower −dT/dt in postrest contraction and after isoproterenol stimulation; and higher time to peak tension after calcium and isoproterenol stimulation in DM-Sed compared to C-Sed. These results allow us to conclude that diabetes-induced systolic and diastolic myocardial dysfunction is responsible for the echocardiographic pattern of dilated cardiomyopathy observed in the* in vivo* evaluation.

The echocardiographic study revealed that left atrium diameter was lower in DM-Ex than DM-Sed. Left atrium dilation is often caused by an increase in LV diastolic pressure, which may result from alterations in LV diastolic and/or systolic function. In clinical studies, left atrium dimension plays an important role in LV diastolic function evaluation. In fact, an increase in left atrium diameter can be considered an early indicator of diastolic dysfunction [62]. Physical exercise attenuated myocardial dysfunction as DM-Ex had papillary muscle parameter values between those of DM-Sed and C-Ex but not significantly different from either group, except for lower −dT/dt after isoproterenol stimulation than C-Ex. In DM-Ex, the pulmonary congestion marker (wet/dry lung weight ratio) also presented values between those of DM-Sed and C-Ex. We can therefore conclude that exercise attenuated lung congestion, left atrium dilation, and systolic and diastolic myocardial dysfunction in diabetic rats. Physical exercise has been previously shown to improve cardiac function in diabetic rats and attenuate diabetes-induced impaired fractional shortening, contraction velocity, and relaxation time in isolated cardiomyocytes [55, 63].

In diabetes, several myocardial changes can be involved in myocardial dysfunction. In this study, we evaluated myocyte hypertrophy, myocardial fibrosis, and oxidative stress as mechanisms potentially involved in the benefits of physical exercise.

Myocardial fibrosis was assessed by measuring myocardial concentration of hydroxyproline, an amino acid, and the main component of the collagen molecule which can only be found in small concentrations in a limited number of other proteins. We observed no difference in hydroxyproline concentration between groups. Myocyte hypertrophy, assessed by measuring the lower transverse diameter, also did not differ between groups. We can therefore conclude that myocardial fibrosis and myocyte hypertrophy are not involved in the cardiac effects of physical exercise. Some authors have previously observed increased myocardial interstitial collagen in streptozotocin-induced diabetes rats [33, 64, 65] and attenuation by physical exercise [33, 65]. Different data can result from the different methods used to evaluate myocardial fibrosis such as ultrastructural analysis [33], protein expression measurement by western blot technique [64], or morphometric analysis using Sirius-red staining [65].

We next evaluated myocardial oxidative stress status. Diabetes increased oxidative stress as lipid hydroperoxide concentration was higher and the activity of antioxidant enzymes superoxide dismutase, catalase, and glutathione peroxidase was lower in DM-Sed than C-Sed. Increased myocardial oxidative stress has previously been reported in diabetic rats [59, 66]. We have previously shown that myocardial oxidative stress in aged diabetic spontaneously hypertensive rats was associated with impaired myocardial and ventricular function [9]. Oxidative stress is a state of imbalance between reactive oxygen species production and endogenous antioxidant capacity [67]. Our data showed that diabetes induced both increased reactive oxygen species production and decreased antioxidant capacity due to reduced antioxidant enzymes activity.

Oxidative stress is a common underlying mechanism in many diseases including cardiovascular diseases. Current knowledge indicates that oxidative stress plays a role as a major determinant of the onset and progression of diabetes-associated cardiovascular alterations [3, 8, 10]. Although it has been established that oxidative stress plays a major role in the development of cardiovascular diseases, clinical studies have failed to show improved outcomes after antioxidant supplementation in preventing or treating cardiovascular diseases [67, 68]. While the mechanisms responsible for these poor results are not clear, researchers have directed their attention towards nonpharmacological therapy to reduce oxidative stress, with physical exercise attracting great interest. Recent experimental studies have shown that chronic moderate exercise can decrease oxidative stress in skeletal muscles [69, 70]. In this study, we observed that physical exercise prevented an increase in myocardial lipid peroxidation and attenuated a decrease in antioxidant enzymes activity. Additional studies are needed to elucidate the mechanisms involved in exercise-induced decrease in myocardial oxidative stress during diabetes mellitus.

Studies have shown that increased oxidative stress jeopardizes myocardial function through mechanisms such as microvascular damage, abnormalities in calcium homeostasis, and endothelial dysfunction [11]. Although a causative role could not be demonstrated in this study, myocardial oxidative stress may have been involved in the exercise-induced improvement in myocardial function and cardiac remodeling in diabetes rats. Our data therefore support the idea of the beneficial role of low intensity exercise in myocardial function and cardiac remodeling in rats with unmanaged type 1 diabetes mellitus.

In conclusion, low intensity physical exercise attenuates myocardial oxidative stress, lung congestion, left atrium dilation, and myocardial dysfunction in type 1 diabetic rats.

## Figures and Tables

**Table 1:** Anatomical data.

	C-Sed (*n* = 13)	C-Ex (*n* = 12)	DM-Sed (*n* = 12)	DM-Ex (*n* = 16)
Final BW	492 ± 28	467 ± 36^*^	390 ± 60^*^	356 ± 50^#§^
LV (g)	0.89 ± 0.07	0.86 ± 0.06	0.74 ± 0.12^*^	0.69 ± 0.10^#^
LV/BW (g/kg)	1.84 ± 0.11	1.91 ± 0.17	2.10 ± 0.21^*^	2.18 ± 0.21^#^
RV (g)	0.22 ± 0.07	0.20 ± 0.02	0.18 ± 0.02^*^	0.16 ± 0.04^#^
RV/BW (g/kg)	0.45 ± 0.15	0.45 ± 0.06	0.51 ± 0.09	0.51 ± 0.12
Atria (g)	0.09 ± 0.01	0.10 ± 0.01	0.09 ± 0.01	0.08 ± 0.01^#§^
LV wet/dry	3.94 (3.56–4.11)	3.60 (3.17–4.17)	4.06 (3.92–4.61)	3.70 (2.84–4.53)
RV wet/dry	3.42 ± 0.65	3.60 ± 0.94	3.96 ± 1.02	3.50 ± 0.77
Lung wet/dry	4.55 ± 0.50	4.56 ± 0.35	4.94 ± 0.58^*^	4.70 ± 0.26

Values are mean ± SD or median and 25th and 75th percentiles. C-Sed: sedentary control group; C-Ex: exercised control group; DM-Sed: sedentary diabetic group; DM-Ex: exercised diabetic group; LV: left ventricle weight; BW: body weight; RV: right ventricle weight; wet/dry: wet-to-dry weight ratio. ANOVA and Bonferroni or Kruskal-Wallis and Dunn; ^∗^ *P* < 0.05 versus C-Sed; ^#^ *P* < 0.05 versus C-Ex; ^§^ *P* < 0.05 versus DM-Ex.

**Table 2:** Echocardiographic data.

	C-Sed (*n* = 14)	C-Ex (*n* = 13)	DM-Sed (*n* = 12)	DM-Ex (*n* = 14)
HR (bpm)	276 ± 11	285 ± 21	290 ± 21	281 ± 20
LVDD (mm)	8.52 ± 0.32	8.13 ± 0.47^*^	8.47 ± 0.52	8.26 ± 0.33
LVDD/BW (mm/kg)	17.4 (16.8–17.9)	17.5 (16.5–19.1)	23.2 (19.6–25.1)^*^	23.9 (22.2–24.8)^#^
LVSD (mm)	4.16 ± 0.52	3.97 ± 0.40	4.26 ± 0.59	4.15 ± 0.50
LVPWT (mm)	1.41 ± 0.05	1.45 ± 0.07	1.49 ± 0.16	1.44 ± 0.15
LVMI (g/kg)	1.77 (1.74–1.90)	1.84 (1.70–2.03)	2.43 (2.10–2.75)^*^	2.38 (2.26–2.60)^#^
LVRWT	0.33 ± 0.02	0.36 ± 0.02^*^	0.35 ± 0.04	0.35 ± 0.04
LA (mm)	5.73 ± 0.49	5.67 ± 0.53	6.41 ± 0.54^*^	5.81 ± 0.50^§^
LA/AO	1.45 ± 0.12	1.49 ± 0.12	1.74 ± 0.12^*^	1.60 ± 0.03^#§^
EFS (%)	51.2 ± 5.14	51.3 ± 2.98	49.9 ± 4.55	49.8 ± 5.33
EF	0.88 ± 0.04	0.88 ± 0.02	0.87 ± 0.03	0.88 ± 0.04
PWSV (mm/s)	38.8 ± 4.02	39.4 ± 4.54	34.1 ± 2.85^*^	35.7 ± 4.52^#^
E/A	1.53 ± 0.22	1.65 ± 0.32	1.35 ± 0.22	1.48 ± 0.24
EDT (ms)	47.4 ± 5.79	47.6 ± 6.70	44.7 ± 6.07	47.4 ± 8.71
Tei index	0.48 ± 0.06	0.52 ± 0.08	0.52 ± 0.05	0.58 ± 0.10

Values are mean ± SD or median and 25th and 75th percentiles. C-Sed: sedentary control group; C-Ex: exercised control group; DM-Sed: sedentary diabetic group; DM-Ex: exercised diabetic group; HR: heart rate; LVDD and LVSD: left ventricular (LV) diastolic and systolic diameters, respectively; BW: body weight; LVPWT: LV posterior wall thickness; LVMI: LV mass index; LVRWT: LV relative wall thickness; LA: left atrium diameter; AO: aorta diameter; EFS: LV endocardial fractional shortening; EF: LV ejection fraction; PWSV: LV posterior wall shortening velocity; E/A: ratio of early (E wave) to late (A wave) diastolic mitral inflow velocities; EDT: E wave deceleration time. ANOVA and Bonferroni or Kruskal-Wallis and Dunn; ^∗^ *P* < 0.05 versus C-Sed; ^#^ *P* < 0.05 versus C-Ex; ^§^ *P* < 0.05 versus DM-Ex.

**Table 3:** Isolated papillary muscle data at basal condition and after positive inotropic stimulation.

		C-Sed (*n* = 14)	C-Ex (*n* = 13)	DM-Sed (*n* = 12)	DM-Ex (*n* = 14)
	DT (g/mm^2^)	8.57 ± 2.13	7.13 ± 1.82	7.35 ± 1.13	7.23 ± 1.53
	TPT (ms)	169 ± 15	175 ± 15	183 ± 16	176 ± 16
Basal	+dT/dt (g/mm²/s)	99.9 ± 29.1	78.9 ± 17.0^*^	78.9 ± 16.8^*^	76.6 ± 17.4
	−dT/dt (g/mm²/s)	32.2 (29.8–42.6)	29.8 (25.3–37.0)	27.1 (23.8–33.6)	28.2 (24.2–35.7)
	RT (g/mm^2^)	1.02 (0.81–1.09)	0.90 (0.66–1.04)	0.88 (0.75–0.97)	0.88 (0.70–0.97)

	DT (g/mm^2^)	10.56 ± 2.37	8.68 ± 2.59	8.45 ± 1.56^*^	8.45 ± 1.96
	TPT (ms)	160 (160–180)	180 (170–193)	180(170–190)	170 (170–193)
PP30	+dT/dt (g/mm²/s)	122 ± 32.9	94.5 ± 25.6^*^	90.8 ± 21.1^*^	88.8 ± 21.2
	−dT/dt (g/mm²/s)	39.1 ± 12.2	34.1 ± 9.14	29.4 ± 6.88^*^	30.4 ± 7.14
	RT (g/mm^2^)	0.98 (0.84–1.09)	0.91 (0.56–1.04)	0.89 (0.75–0.94)	0.89 (0.69–1.05)

	DT (g/mm^2^)	8.97 (8.35–10.6)	8.80 (7.56–12.1)	7.73 (6.58–9.07)	8.97 (6.20–11.3)
	TPT (ms)	160 (160–180)	170 (170–180)	195 (170–200)^*^	170 (160–180)
2.5 mM [Ca^2+^]_0_	+dT/dt (g/mm²/s)	119 ± 28.4	108 ± 36.0	90.3 ± 22.9	84.6 ± 37.2
	−dT/dt (g/mm²/s)	36.2 (32.4–46.5)	38.3 (31.4–55.8)	32.0 (25.7–38.6)	24.6 (20.8–39.9)
	RT (g/mm^2^)	0.89 ± 0.30	0.72 ± 0.34	0.68 ± 0.19	0.76 ± 0.35

	DT (g/mm^2^)	7.80 (7.08–9.86)	6.71 (5.53–9.48)	7.14 (5.95–8.46)	6.91 (6.45–8.81)
	TPT (ms)	149 ± 13	160 ± 17	167 ± 9^*^	167 ± 14
10^−6^ M Iso	+dT/dt (g/mm²/s)	117 ± 25.6	99.5 ± 25.1	92.7 ± 22.1^*^	91.1 ± 17.7
	−dT/dt (g/mm²/s)	58.1 ± 11.5	55.3 ± 13.6	44.4 ± 12.4^*^	44.3 ± 9.67^#^
	RT (g/mm^2^)	0.85 ± 0.32	0.60 ± 0.31	0.65 ± 0.18	0.73 ± 0.34

Values are mean ± SD or median and 25th and 75th percentiles. C-Sed: sedentary control group; C-Ex: exercised control group; DM-Sed: sedentary diabetic group; DM-Ex: exercised diabetic group; DT: peak of developed tension; TPT: time to peak of tension; +dT/dt: maximum rate of tension development; −dT/dt: maximum rate of tension decline; RT: resting tension. Basal: isometric contraction with 1.25 mM extracellular calcium concentration; PP30: postrest contraction of 30 s; 2.5 mM [Ca^2+^]_0_: isometric contraction with 2.5 mM extracellular calcium concentration; 10^−6^ M Iso: isometric contraction with 10^−6^ M isoproterenol added to the nutrient solution. ANOVA and Bonferroni or Kruskal-Wallis and Dunn; ^∗^ *P* < 0.05 versus C-Sed; ^#^ *P* < 0.05 versus C-Ex.
